# Photocurrents from photosystem II in a metal oxide hybrid system: Electron transfer pathways

**DOI:** 10.1016/j.bbabio.2016.03.004

**Published:** 2016-09

**Authors:** Katharina Brinkert, Florian Le Formal, Xiaoe Li, James Durrant, A. William Rutherford, Andrea Fantuzzi

**Affiliations:** a Department of Life Sciences, Imperial College London, London SW7 2AZ, UK; b Department of Chemistry, Imperial College London, London SW7 2AZ, UK

**Keywords:** *β-DDM*, n-Dodecyl β-d-maltoside, *DCBQ*, 2,6-dichloro-1,4-benzoquinone, *DCMU*, 3-(3,4-dichlorophenyl)-1,1-dimenthylurea, *E*_*f*_, energetic position of the Fermi level, *E*_*m*_, midpoint potential, *E*_*V*_, energetic position of the valence band, *E*_*c*_, energetic position of the conduction band, *K*_*d*_, binding constant, *K*_*m*_, Michaelis–Menten constant, *MES*, 2-(N-morpholino)ethane-sulfonic acid, *ITO*, indium tin oxide, *TiO*_*2*_, titanium dioxide, *NiNTA*, Ni^2 +^-nitrilotriacetic acid, *PSII*, Photosystem II, *PpBQ*, 2-phenyl-*p*-benzoquinone, *SHE*, standard hydrogen electrode, *TMPD*, N,N,N′,N′-tetramethyl-p-phenylenediamine, *v*/*v*, volume/volume, volume concentration, *V*_*FB*_, flat band potential, *w*/*v*, mass/volume, mass concentration, Water oxidizing enzyme, Photosynthetic reaction centre, Photosynthesis, Protein electrode interface, Protein film photoelectrochemistry, Quinone

## Abstract

We have investigated the nature of the photocurrent generated by Photosystem II (PSII), the water oxidizing enzyme, isolated from *Thermosynechococcus elongatus*, when immobilized on nanostructured titanium dioxide on an indium tin oxide electrode (TiO_2_/ITO). We investigated the properties of the photocurrent from PSII when immobilized as a monolayer *versus* multilayers, in the presence and absence of an inhibitor that binds to the site of the exchangeable quinone (Q_B_) and in the presence and absence of exogenous mobile electron carriers (mediators). The findings indicate that electron transfer occurs from the first quinone (Q_A_) directly to the electrode surface but that the electron transfer through the nanostructured metal oxide is the rate-limiting step. Redox mediators enhance the photocurrent by taking electrons from the nanostructured semiconductor surface to the ITO electrode surface not from PSII. This is demonstrated by photocurrent enhancement using a mediator incapable of accepting electrons from PSII. This model for electron transfer also explains anomalies reported in the literature using similar and related systems. The slow rate of the electron transfer step in the TiO_2_ is due to the energy level of electron injection into the semiconducting material being below the conduction band. This limits the usefulness of the present hybrid electrode. Strategies to overcome this kinetic limitation are discussed.

## Highlights

• In PSII metal oxide hybrid systems, direct electron transfer occurs from Q_A_ to the electrode surface.
• Electron transfer through the nanostructured TiO_2_ is rate-limiting.
• Redox mediators can overcome the limitation by taking electrons from the TiO_2_ semiconductor to the electrode surface.

## Graphical abstract

**Figure:**
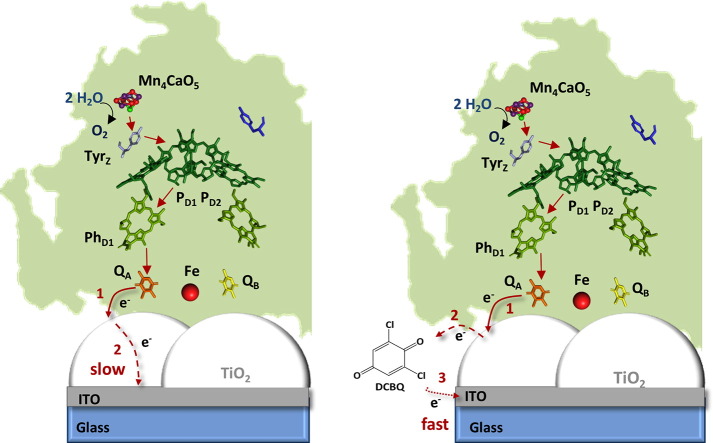

## 1 Introduction

**Fig. 1:**
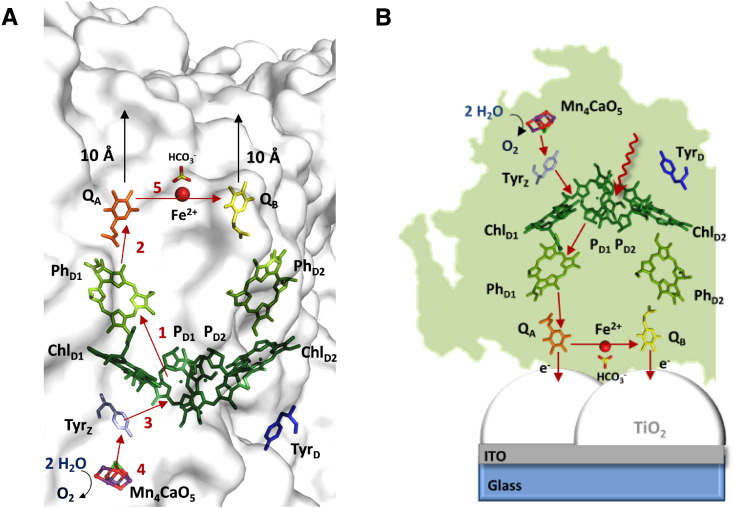
(A) Schematic representation of the arrangement of cofactors involved in the electron transfer chain in Photosystem II based on the 1.95 Å crystal structure (PDB reference 4UB6) [6]. The numbers represent the order of electron transfer steps after charge separation. Step 1 represents both the charge separation and the first stabilization step (see text) forming the radical pair. The black arrows indicate potential exit routes for electrons from the quinones Q_A_ and Q_B_ to the protein surface. (B) Scheme of the orientation of PSII on the TiO_2_/ITO electrode and indication of the electron transfer steps after charge separation. The two possibilities of electron transfer from the enzyme to the electrode are indicated.

The conversion of solar energy into chemical energy through oxygenic photosynthesis is one of the most important biological processes. The key reaction is the light-driven oxidation of water, which occurs in Photosystem II (PSII) [1], [2], [3], [4]. PSII is a large, multi-subunit trans-membrane protein complex, which contains pigments and cofactors and is found in the photosynthetic membranes of cyanobacteria and photosynthetic eukaryotes [1], [2], [3], [4], [5], [6]. Photoexcitation of chlorophylls in PSII initially generates a distribution of radical pairs. Rapid electron transfer reactions produce a secondary radical pair that consists of the cation radical localized on the chlorophyll known as P_D1_ and the anion radical localized mainly on the pheophytin, Ph_D1_ (Fig. 1) [1], [2], [3]. Electron transfer from the pheophytin anion radical, Ph_D1_^−^•, reduces a bound plastoquinone, Q_A_, forming the semiquinone anion radical, Q_A_^−^•. The electron on Q_A_^−^• is transferred to a second quinone, Q_B_, which is exchangeable when oxidized or fully reduced and is tightly bound only when in the Q_B_^−^• state. When a second light-induced charge separation takes place, Q_B_^−^• becomes protonated forming the quinol Q_B_H_2_, which then exchanges for another quinone in the membrane pool. The electron hole at P_D1_^+^• is able to oxidize a tyrosine residue, Tyr_Z_. The neutral tyrosyl radical, Tyr_Z_• oxidizes a heteronuclear Mn_4_CaO_5_ cluster located on the luminal side of the enzyme. When four successive charge equivalents are accumulated on the cluster (each state known as an S-state), the metal cluster oxidizes two molecules of water with the release of O_2_ and four protons [1], [2], [4].

Knowledge of PSII has inspired the field of artificial photosynthesis, in which robust and cheap catalysts are being developed for the photochemical and electrochemical generation of fuels using solar energy [7], [8], [9], [10], [11], [12], [13].

The enzyme itself is often considered to have applications in a range of photoelectrochemical devices [14], [15], [16], [17], [18]. However, the use of isolated PSII in energy generation appears unrealistic, not only because of the energy, time and effort required for isolating it from the living cell, but also because PSII undergoes photodamage. Indeed the D_1_ protein, which is the location of the damage, is the most rapidly turned-over protein in the thylakoid membrane [19]. Its degradation strongly depends on the incident light intensity and it can have a half-life of only 30 min [20]. Nevertheless, applications of isolated PSII that do not require the scale or longevity needed for energy production are conceivable (*e.g.* sensors for pollutants and herbicides [21], [22]). Additionally, the utilization of PSII in devices could become advantageous if a new form of PSII with enhanced photostability is either engineered or isolated from an organism living in extreme conditions [23].

PSII is a particularly interesting system for studying electron transfer from protein-bound cofactors to electrode materials since it is the only reaction center capable of taking electrons from water and thus it does not need electron donors that could react with the electrode directly. A well understood electronic coupling between PSII and an electrode surface could allow an additional avenue of research on the enzyme itself.

Photocurrents from PSII immobilized onto electrode surfaces have been studied for decades (for example [14], [15], [16], [17], [18], [24], [25], [26], [27], [28], [29], [30], [31], [32], [33], [34], [35] for a complete review of the most recent state of knowledge see [14], [15], [16], [17], [18]). Given the crystal structures [5], [6], [36], [37], [38], it is now clear that electron transfer between cofactors in the enzyme and the electrode seems feasible from three cofactors that are located close enough to the periphery to allow electron transfer to a conductor (or electron acceptor) in contact with the surface: Q_A_, Q_B_ and Fe, all of which are on the PSII electron acceptor side of the protein (Fig. 1) [6]. The iron is slow to undergo oxidation and does not undergo redox reactions under the normal electron transfer conditions. In solution, electron transfer from Q_A_^−^ to soluble electron acceptors at the protein surface was reported when the Q_B_-site was blocked by the urea herbicide DCMU [39], [40], [41], [42]. Direct electron transfer from Q_A_ to electrode surfaces was proposed to explain the partial insensitivity of photocurrent to Q_B_ site inhibitors [30], [31].

There are potentially two different ways for the electrons to reach the electrode: 1) directly from Q_A_, Q_B_ and potentially the non-heme Fe and 2) indirectly *via* exogenous electron acceptors (mediators), which transfer electrons from the reduced quinones, mainly from the Q_B_ site, to the electrode surface [14], [15], [16]. Enhanced, direct (non-mediated) photocurrents were observed by orienting the PSII complexes with the acceptor-side towards the electrode surface either by immobilizing His-tagged PSII on Ni^2 +^-nitrilotriacetic acid (NiNTA) modified gold surfaces [16], [28] or by using dipole effects and electrostatic interactions on both un-modified and self-assembled monolayer modified indium tin oxide (ITO) electrodes [14], [31]. The addition of artificial electron acceptors such as 2,6-dichloro-1,4-benzo-quinone (DCBQ) results in a large increase in photocurrent, although the overall magnitude differs substantially in different reports [14], [15], [16].

The enhancement of photocurrent suggests that the electron acceptor acts as a mobile mediator carrying electrons from the reduced quinone cofactors in PSII to the electrode surface. The enhancement is expected to occur by allowing electron transfer from any PSII that is unable to undergo direct electron transfer to the electrode, *i.e.* when i) PSII particles in the contact layer are bound with an orientation in which the quinones are too far from the electrode and ii) when PSII is not in the contact layer, *i.e.* when multilayers of PSII exist.

Anomalous results with the photocurrents from PSII indicate that the present understanding of the reactions occurring is incomplete. In particular, on metal oxides, the addition of the herbicide DCMU, which is expected to shut down the mediated electron transfer by competing with exogenous quinone acceptors at the Q_B_ site, resulted in significant residual photocurrents which could not be accounted for by direct electron transfer from Q_A_^−^• in the contact layer [27], [30]. Until now efforts have been focused mainly on the phenomenon of the photocurrent itself and its maximization. However, the characterization of the electron pathway from the protein to the electrode and understanding the involvement of mobile mediators are both important for developing this methodology, for understanding the enzyme and for any potential applications.

Little if any work has been done on characterizing the electron pathway from the protein to the electrode and the role of the mobile mediators. The focus on obtaining maximum photocurrents has led to the use of protein multilayers (in the presence of mediators) but this gives rise to heterogeneity, with direct and mediated electron transfer to the electrode potentially occurring at the same time.

Here we have controlled the PSII layer thickness on the electrode surface working with a monolayer/sub-monolayer and with multilayers of PSII. Work with the monolayer inevitably results in much smaller photocurrents but it allows much less ambiguous results than working with multilayers. We investigated the effect of the herbicide DCMU and redox mediators on the photocurrents generated with electrodes using monolayers and multilayers of PSII. We also changed the electrode structure to control access to the ITO. The results allow us to propose a new model for the electron transfer in this kind of PSII/metal oxide hybrid system.

## 2 Materials and methods

### 2.1 Materials

3-(3,4-Dichlorophenyl)-1,1-dimethylurea (DCMU), 2,6-dichloro-1,4-benzo-quinone (DCBQ, E _m_ = + 319 mV *vs* SHE, pH 7, determined *via* cyclic voltammetry in a three electrode system with a platinum mesh working electrode, a platinum counter electrode and an Ag/AgCl reference) and 2-(3,4-dihydroxyphenyl)-3,5,7-trihydroxy-4H-1-benzopyran-4-on (quercetin, E_m_ = + 331 mV *vs* SHE, pH 7, determined as described above for DCBQ), phenyl-*p*-benzoquinone (P*p*BQ) (E_m_ = + 279 mV *vs* SHE, pH 7 [43]) and additional chemicals were purchased from Sigma Aldrich. Nanostructured TiO_2_ on conductive ITO glass were obtained from Solaronix S. A., Aubonne, Switzerland (20 nm particle size, 250–500 nm layer thickness) and used as electrodes for photocurrent generation.

In some of the experiments carried out to investigate the role of mediators, electrodes were used in which a thin layer of crystalline TiO_2_ separated the nanostructured TiO_2_ from the conducting ITO glass. The thin layer (100 nm thickness) of TiO_2_ was prepared by spray pyrolysis according to Oja et al. (2004) [44] followed by the deposition of the nanostructured TiO_2_. The thickness of the insulating layer of crystalline TiO_2_ is such that electron transfer still occurs between the mesoporous TiO_2_ and the ITO while electron transfer from any freely diffusing molecule is strongly inhibited.

### 2.2 Isolation and characterization of PSII core complexes

Photosystem II core particles were isolated from a CP47 His-tagged mutant from the thermophylic cyanobacterium *Thermosynechococcus elongatus* BP-1 by Ni^2 +^-affinity chromatography as described by Sedoud et al. [45] using a protocol based on Sugiura and Inoue [39] with the same buffers but with the following additional modifications: *T. elongatus* were grown in temperature regulated orbital shakers in 5 L Erlenmeyer flasks to a volume of 3 L. In total 18 L was cultured in DTN medium, supplemented with 10 mM of bicarbonate at 45 °C in a rotary shaker (120 rpm) and a light intensity of 40 μE m^− 2^·s^− 1^. When the optical density at 730 nm reached ~ 1.0 the cells were harvested using a cell concentrator pump (Watson-Marlow Pumps Group), followed by centrifugation and washing in Buffer 1 (40 mM MES (pH 6.5), 15 mM MgCl_2_, 15 mM CaCl_2_, 10% (*v*/v) glycerol, 1.0 M betaine). The cells were ruptured by passing the suspension twice through a chilled Cell Disruptor (Constant Systems, Model T5) at 25 kpsi. Samples were kept in near darkness and at 4 °C. The crude extract was spun down at 5000 ×* g* for 5 min to pellet cell debris. The supernatant was loaded on to a Ni^2 +^-affinity chromatography column as described in Sedoud et al. [45]. The eluted PSII core complexes were concentrated using centrifugal filter tubes (Amicon Ultra) with a molecular weight cut-off of 100,000 NMWL spun at 4000 ×* g* until most of the Buffer 3 (40 mM MES (pH 6.5), 15 mM MgCl_2_, 15 mM CaCl_2_, 200 mM NaCl, 300 mM imidazole, 0.06% (*w*/*v*) β-DDM, 10% (*v*/v) glycerol, 1.0 mM betaine) had passed through and then washed three times with Buffer 1. The PSII was finally concentrated to a chlorophyll a concentration of ~ 3 mg·mL^− 1^ and stored in Buffer 1 (storage buffer) in liquid nitrogen.

Oxygen evolution activity was assayed with a Clark-type oxygen electrode (Oxylab, Hansatech) at 25 °C in the presence of 0.5 mM of DCBQ and 1.0 mM of potassium ferricyanide, using saturating red light (590 nm cut-off filter; 13,000 μE·m^− 2^·s^− 1^). The activity in the various preparations were about 3500 μmol O_2_·mg Chl a^− 1^·h^− 1^ under these conditions.

### 2.3 Electrode preparation and immobilization of PSII

The method for protein immobilization was derived from previous studies [46], [47], [48]. Electrodes were heated at 450 °C for 10 min and cooled to room temperature before use. 40 μL of PSII solution in the storage buffer containing 0.03% β-DDM with either a chlorophyll a concentration of 4 μg/mL or 400 μg/mL (for studies of monolayers or multilayers of PSII, respectively) were used to cover the electrode surface. It was found empirically that the lowest amount of PSII needed to give a measurable direct photocurrent was approximately 2 μg/mL, thus double that concentration was chosen to provide an adequate signal to noise ratio. The concentration for multilayers was chosen to be 100 times higher. PSII immobilization onto the electrode was allowed to occur in a water-saturated atmosphere in the dark at 4 °C overnight. Before the measurements, the electrode was rinsed with ultrapure water to remove non-immobilized PSII and placed in a vessel containing the electrolyte buffer used for the electrochemical measurements. The vessel was kept in the dark on ice prior to the measurement. The final amount of PSII on the TiO_2_ surface was determined by quantifying the amount of chlorophyll a using a NanoDrop Spectrophotometer (Thermo Scientific, Nano-Drop 1000). Chlorophyll was extracted from PSII on the electrode surface with 40 μL methanol (99.9%). The amount of chlorophyll a was determined by measuring its absorption in methanol at 665 nm using an extinction coefficient of 79.95 mg·mL^− 1^·cm^− 1^ [39], correcting for volume changes occurring during extraction. The amount of PSII was deduced based on 35 chlorophylls/PSII [6]. Taking into account the size of the PSII monomers (approximately 10^− 12^ cm^2^ based on the crystal structure [38]) and the roughness of the TiO_2_ surface, the accessible area was estimated to be about 4–5 times that of the geometrical area of the TiO_2_ layer. Based on this estimate the amount of PSII on the surface was found to be 1.2 pmol cm^− 2^ when the low chlorophyll concentration (4 μg/mL) was used, corresponding to the formation of a monolayer/sub-monolayer on the electrode surface. Confocal fluorescence microscopy shows that samples prepared using protein concentration of 4 μg/mL present unaltered morphology compared to a control without protein while showing a uniform fluorescence signal across the electrode surface. These results are consistent with the formation of a uniform monolayer when using 4 μg/mL. Furthermore, according to Kato et al. [31], in these experimental conditions the electrostatic interaction between the protein and the electrode surface, guided by the protein electric dipole, would orient the protein with the protein-bound quinones facing towards the electrode. The fact that the PSII is in a (sub)-monolayer, in which any excess unbound PSII is washed away, will favor immobilization of only those centers that are tightly bound, *i.e.* those with the acceptor side (the electrostatically favored side) facing the electrode. Additionally, if we consider the porous nature of the TiO_2_ surface, the immobilized proteins are predicted to be located in the TiO_2_ cavities surrounded in large part by the electrode material, like eggs in an egg box. It is therefore likely that in the monolayer most of the protein-bound quinones are within electron transfer distance from the electrode surface.

### 2.4 Electrochemistry

Electrochemical measurements were carried out using a PGSTAT12 electrochemical analyzer controlled by GPES software (Eco Chemie Utrecht).

An open glass cell was used with a platinum wire as a counter electrode and a saturated calomel electrode (SCE) as a reference. An external bias of + 644 mV *vs* SHE (if not indicated otherwise) was applied before each measurement for 250 s to let the system equilibrate in the dark. In order to minimize the photodegradation of the immobilized PSII, the photocurrents were recorded using short illumination intervals. The length of each illumination period was chosen according to the time needed to obtain a stable photocurrent. In the absence of any external mediator a stable photocurrent was reached within 10 s, while in the presence of an external mediator, due to diffusion controlled phenomena, illumination periods of 20 s were used. Each illumination interval was followed by a period of 60–100 s in the dark. The photocurrent was considered to have reached equilibrium when two subsequent illumination intervals showed the same current values within the standard deviation. In both cases, with and without external mediator, the photocurrent reached equilibrium after 60 s. All photocurrent values and traces presented in the manuscript are at equilibrium, if not stated otherwise. All measurements were carried out at 25 °C. An electrolyte buffer solution containing 20 mM of CaCl_2_, 40 mM of MES and 5% glycerol at pH 6.5 (if not indicated otherwise) was used. 10 mM stock solutions of DCBQ and quercetin were prepared in ethanol (99.8%) and 10 mM DCMU stock solutions were prepared in DMSO (99.9%). All redox potentials are *vs* SHE. Continuous illumination was provided by a xenon lamp and the light was filtered through a 590 nm cut-off filter producing red light with an intensity of 800 μE m^− 2^ s^− 1^ in the cell. This light intensity did not induce any detectable photocurrent from the TiO_2_/ITO surface in the absence of PSII.

The error range (n = 4) for photocurrent densities recorded for both, PSII mono- and multilayers, was approximately ± 10 nA/cm^2^ in the absence of mediators. In the presence of mediators, the error range (n = 4) for photocurrent densities recorded of PSII monolayers was approximately ± 20 nA/cm^2^ and for PSII multilayers ± 150 nA/cm^2^.

## 3 Results and discussion

**Fig. 2:**
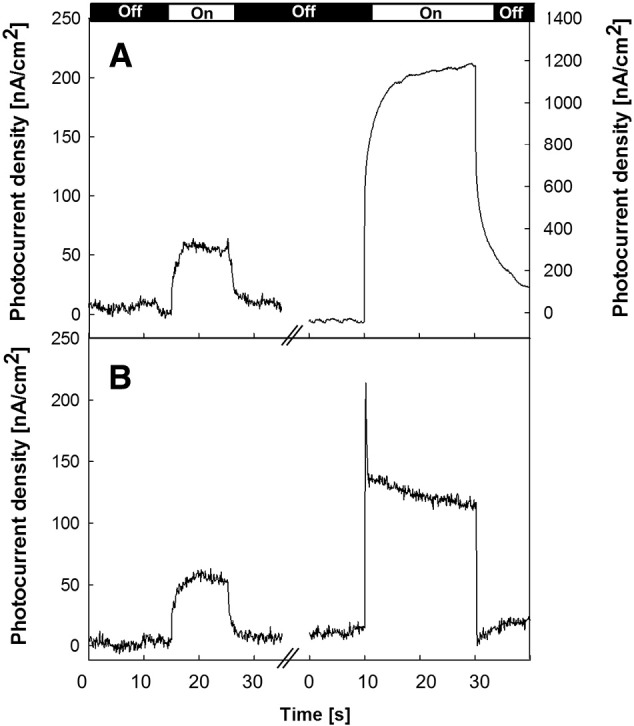
Photocurrent response from PSII multilayers (A) and monolayers (B) adsorbed onto a nanostructured TiO_2_/ITO electrode surface in the absence (left traces) and presence (right traces) of the redox mediator 100 μM DCBQ. Note the bigger scale for right trace in A (multilayers plus mediator). Note: The bar at the top shows the length of the illumination periods, 10 s for the trace on the left and 20 s for the one on the right (see experimental section).

Fig. 2 shows photocurrents recorded from electrodes with PSII present as multilayers (Fig. 2A) and as a monolayer (Fig. 2B). Protein load quantification and monolayer characterized as described in the Materials and methods section. In the absence of the electron acceptor and mobile electron carrier (mediator) DCBQ (Fig. 2 left traces), the photocurrents are similar in amplitude irrespective of whether the PSII is present as monolayer or multilayer. This suggests that it is only the first layer of immobilized protein that directly communicates with the electrode surface.

When DCBQ was present (Fig. 2 right traces), there were marked differences between the mono- and multi-layer (Fig. 2 right traces) in terms of amplitude and kinetic profile. The monolayer showed an almost instant rise of the photocurrent to a maximum with a slower decay to an equilibrium value in the order of 100–150 nA/cm^2^ (Fig. 2B right trace). The multilayer instead showed a slow rise of the photocurrent to a maximum and stable value of about 1200 nA/cm^2^ (Fig. 2 right trace, note the much bigger scale used for the right hand trace in Fig. 2A). The magnitude of the rapid rise in photocurrent observed for the monolayer was found to be dependent on both the concentration of DCBQ and the presence of DCMU, suggesting an involvement of the Q_B_ site. These rapid kinetics are currently being investigated in more detail.

The differences in the magnitude of the DCBQ-enhanced photocurrent, when comparing the monolayer and multilayers, can be explained as follows: the majority of PSII in the multilayer is outside of the contact layer and only contributes to the photocurrent when the mediator is present. Thus, the presence of DCBQ allows the water-splitting reaction to occur by relaying the electrons from the PSII to the electrode. In addition, the slower kinetics in the multilayer is attributed to limitations associated with diffusion of the mediator within the multilayer.

In the monolayer system, the increase in amplitude of the photocurrent induced by the addition of DCBQ might be explained as representing the fraction of PSII in the contact layer in which the protein is oriented in such a way that Q_A_ is unable to donate electrons directly to the TiO_2_. This interpretation is tested below and an alternative explanation is found to be more likely.

**Fig. 3:**
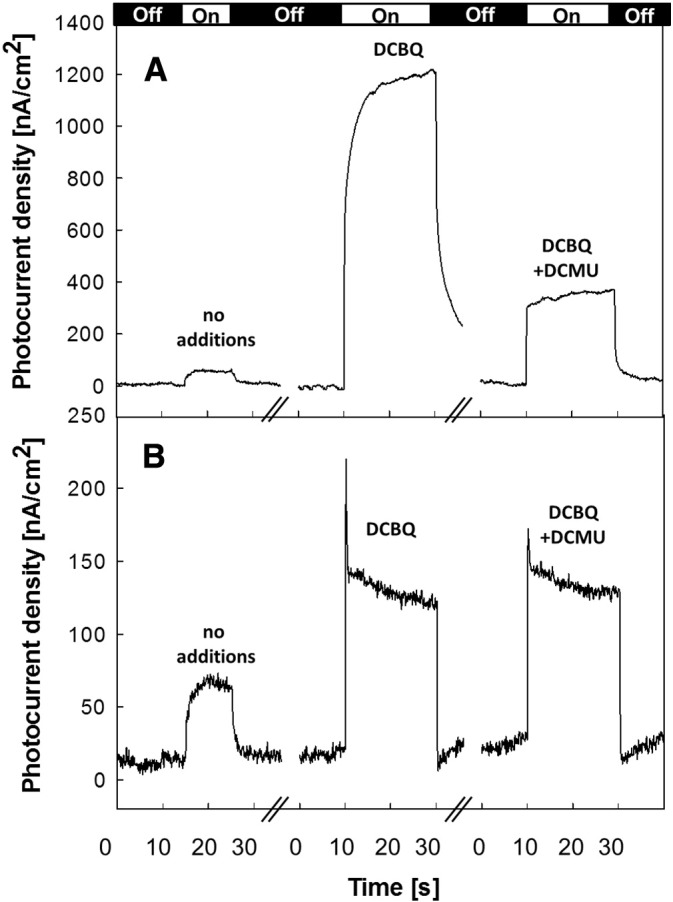
Photocurrent response from PSII multilayers (A) and monolayers (B) adsorbed onto a nanostructured TiO_2_ film in the absence and presence of the mediator DCBQ and the herbicide DCMU. (A) The photocurrent recorded from PSII multilayers (first trace), the presence of 100 μM of DCBQ (second trace) and 10 μM of DCMU (third trace). (B) The photocurrent recorded from a PSII monolayer (first trace) in the presence of 100 μM of DCBQ (second trace) and in the presence of 100 μM DCBQ and 10 μM DCMU (third trace). Note: The bar at the top shows the length of the illumination periods, 10 s for the trace on the left and 20 s for the others (see experimental section).

**Fig. 4:**
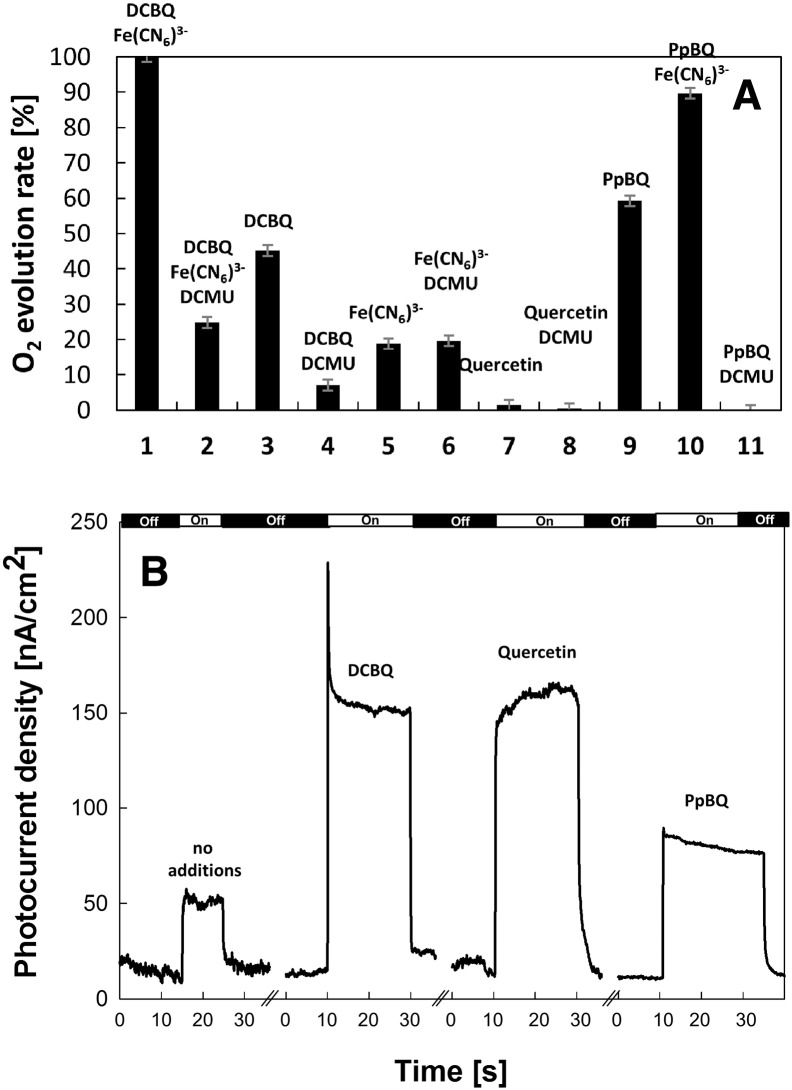
(A) Oxygen evolution measurements of PSII in the presence of 0.5 mM of DCBQ, 1 mM of ferricyanide (1); 0.5 mM of DCBQ, 1 mM of ferricyanide, 50 μM of DCMU (2); 0.5 mM of DCBQ (3); 0.5 mM of DCBQ, 50 μM of DCMU (4); 1 mM of ferricyanide (5); 1 mM of ferricyanide, 50 μM of DCMU (6); 0.5 mM of quercetin (7); 0.5 mM of quercetin, 50 μM of DCMU (8); 0.5 mM of P*p*BQ (9); 0.5 mM of P*p*BQ, 1 mM of ferricyanide (10) and 0.5 mM of P*p*BQ, 50 μM of DCMU (11). Error bars are indicated in gray. (B) Photocurrent response from PSII immobilized onto TiO_2_/ITO electrode as a monolayer; unmediated, in the presence of 100 μM of DCBQ, 100 μM of quercetin or 100 μM of P*p*BQ in the measuring buffer. Note: The bar at the top shows the length of the illumination periods.

Fig. 3 shows the results of experiments comparing the effect of DCMU on the photocurrents generated with a PSII monolayer compared to those with a PSII multilayer. DCMU is a herbicide that works as a Q_B_ site inhibitor, blocking electron transfer from Q_A_^−^• to Q_B_ or to DCBQ in the Q_B_ site. With multilayers of PSII, DCMU produces an incomplete inhibition of the DCBQ-enhanced photocurrent (Fig. 3A). This can only partially be explained by DCBQ accepting electrons from Q_A_^−^• directly (see below and Fig. 4A where this is shown to be 10% of electron transfer). A similar incomplete inhibition of the photocurrent was reported previously by Kato et al. [30]. With the monolayer of PSII, DCMU has no significant effect on the level of the DCBQ-enhanced photocurrent (Fig. 3B).

We tested several possible explanations for the lack of a DCMU effect on the DCBQ enhanced photocurrent when PSII was immobilized as a monolayer.

i) The possibility that DCMU had restricted access to the QB site in the immobilized PSII was tested. PSII was immobilized in the presence of the DCMU. DCBQ addition still induced a comparable enhancement of the photocurrent occurring from the PSII (Figure S1A) indicating that the lack of a DCMU effect is not simply due to restricted access of DCMU to the QB site.
ii) The possibility that DCMU binding affinity (nanomolar [49]) was weaker in the immobilized PSII was tested by increasing the concentration of DCMU. The DCBQ-induced enhancement of photocurrent in the PSII monolayer was unaffected by DCMU up to concentrations of 100 μM (Figure S2). An immobilization induced shift in the binding affinity by several orders of magnitude seems unlikely. Further control experiments showed that a gradual decrease of the photocurrent with time was due to photodamage of the protein and was unrelated to the effect of DCMU.
iii) The possibility that immobilization generates a situation in which DCBQ becomes fixed or trapped between the protein and the TiO_2_ surface was discounted since the enhancement of the photocurrent by DCBQ was reversed when the DCBQ was removed by replacing the buffer (Figure S3).

Based on the experiments described above, it seems that DCBQ and DCMU function as an electron mediator and as a Q_B_ site inhibitor respectively, as expected. The results in Fig. 3 thus indicate that Q_A_^-•^ is able to donate electrons directly to the nanostructured TiO_2_. Two potential mechanisms can be suggested to explain why DCBQ enhances the photocurrent from the PSII monolayer (Fig. 2, Fig. 3) and why DCMU had no effect on the photocurrent under these conditions: i) DCBQ takes electrons directly from Q_A_ and delivers electrons to the TiO_2_ or the ITO; and/or ii) DCBQ takes electrons from the TiO_2_ surface and delivers them to the ITO.

Both mechanisms require the diffusion of DCBQ in solution. This is expected for the mediator but was confirmed by i) the loss of photocurrent when DCBQ is removed from the buffer as mentioned above (Figure S3), and ii) the observation that increasing concentration of DCBQ resulted in a hyperbolic increase in photocurrent with a *K*_*m*_ of 8 μM (Figure S4). This value is however more than 10 times smaller than that measured in oxygen evolution measurements in solution [50], suggesting that the interaction of DCBQ with PSII does not involve the Q_B_ site.

The photocurrent measured as a function of an increasing DCBQ concentration deviated from hyperbolic behavior for concentration values below 1 μM, indicating a threshold below which DCBQ had no or little effect (Figure S4). This seems to suggest that DCBQ competes with another electron transfer route and its effect on the photocurrent can only be observed above a certain concentration in solution. At low DCBQ concentration the slow electron transfer through the metal oxide dominates, while at higher DCBQ concentrations (above 1 μM) the more favorable route provided by the mediator in solution will be preferred.

Fig. 4A shows oxygen evolution measurements of PSII in solution in the presence of different electron acceptors and inhibitors. Maximum oxygen evolution rates are usually measured by using both ferricyanide and DCBQ as electron acceptors. The role of ferricyanide is mainly to re-oxidize the DCBQ that reacted with PSII, accelerating the catalytic reaction, as indicated by the fact that in Fig. 4A, column 1 is larger than the sum of columns 3 and 5. This reflects a situation similar to the one represented by the PSII immobilized on the electrode where the biased electrode re-oxidizes the reduced DCBQ. Therefore all of the measured oxygen evolution rates were presented as a percentage fraction with respect to the value measured with both ferricyanide and DCBQ. Fig. 4A shows that with PSII in solution 10% of the oxygen evolving activity remained when both DCBQ and DCMU were present, in line with previous observations [51]. Assuming that this is due to DCBQ being able to accept electrons from Q_A_^−^• when the Q_B_ site is blocked, this indicates that the rate of electron transfer from Q_A_^−^• to DCBQ is ~ 10 times slower than the electron transfer rate to DCBQ when DCMU is absent. Consequentially the absence of an effect of DCMU on the DCBQ-enhanced photocurrent in the PSII monolayer (Fig. 3B) indicates that this photocurrent cannot be ascribed to DCBQ-mediated electron transfer between PSII and the electrode surface (see below).

The results obtained using the PSII monolayer can be used to analyze the behavior of the PSII multi-layers. For the multilayers of PSII, DCMU should drastically inhibit electron transfer (down to 10%) from PSII in all layers other than the contact layer. The data in Fig. 3 partially fit with this expectation, with the trace from the multilayers of PSII in the presence of DCBQ and DCMU (Fig. 3A right) showing a photocurrent amplitude that is twice that of the monolayer when DCMU is present (Fig. 3B right trace, note the scale difference between A and B).

The smaller amplitude of the photocurrent in the corresponding “monolayer” under these conditions is either due to the reduced DCBQ mediated electron flow from Q_A_^−^• or to the fact that the monolayer is incomplete, while the contact layer at the base of the multilayers is expected to be complete. Nevertheless, the slow rising kinetic, which is characteristic of the DCBQ-enhanced photocurrent in the multilayers of PSII (Fig. 2A right, Fig. 3A middle), is eliminated by DCMU (Fig. 3A right) leading to a photocurrent kinetic profile that is more similar to that of the monolayer. These changes in the kinetic profile suggest that the direct electron transfer from Q_A_ to DCBQ, which in solution is slow (see below), does not play a significant role in the electron transfer process to the electrode also in the multi-layers.

Fig. 4A also shows the oxygen evolution activity in solution with P*p*BQ, another commonly used electron acceptor with PSII [39]. The activity was eliminated when DCMU was present (Fig. 4A, bar 11). Clearly P*p*BQ is unable to accept electrons from Q_A_^−^• and yet P*p*BQ did enhance the photocurrent just as did DCBQ, though with smaller magnitude (Fig. 4B), and this enhancement was also unaffected by DCMU (data not shown). We conclude that the P*p*BQ-enhanced photocurrent does not involve electron transfer from Q_A_^−^• to the mediator. Note, in Fig. 4B the differences in the magnitude of the photocurrents for the different mediators are likely to be due to differences in the reduction potentials (~ 50 mV) and/or their different affinities for the TiO_2_ surface.

The results presented in Fig. 4A and Fig. 4B and described in the previous paragraphs argue strongly against a mechanism in which the mediator takes electrons directly from Q_A_^−^• and delivers them to the TiO_2_ or the ITO. In the following we describe experiments designed to test the alternative mechanism: the mediator taking electrons from the TiO_2_ surface and delivering them to the ITO.

Fig. 4B also shows an experiment using the redox mediator quercetin instead of DCBQ. Quercetin was chosen since it has a similar reduction potential to DCBQ (see Materials and methods), but does not act as an electron acceptor from PSII, as demonstrated in the oxygen evolution experiments shown in Fig. 4A. Fig. 4B shows that quercetin gives rise to an enhancement of the photocurrent, similar to DCBQ and P*p*BQ. This enhancement also occurs in the presence of DCMU.

**Fig. 5:**
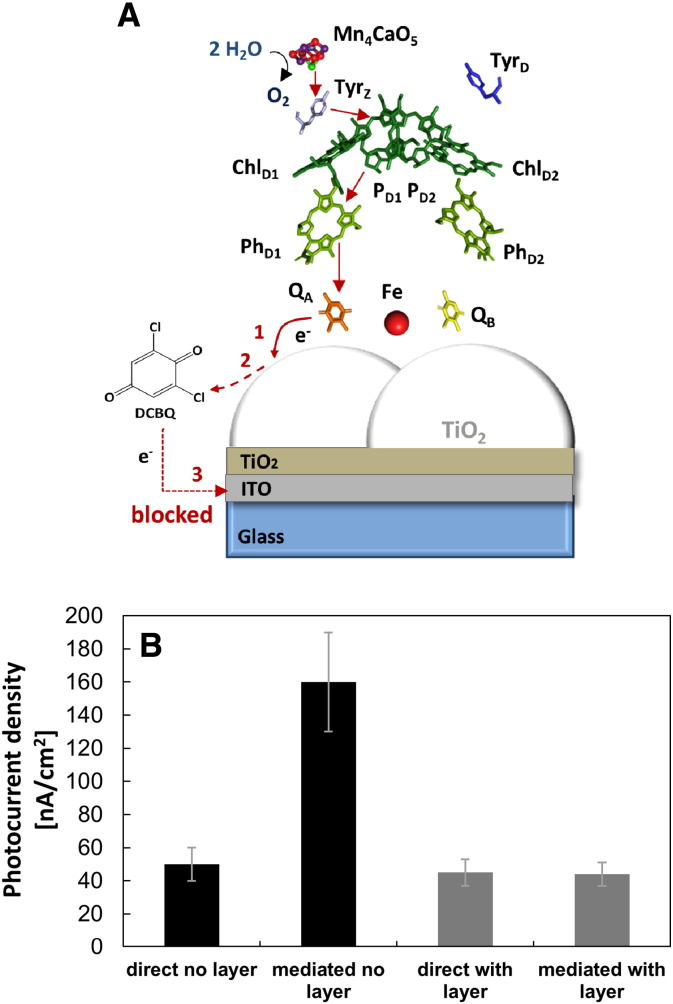
(A) Model illustrating the DCBQ-mediated electron transfer from PSII to ITO when a blocking layer of crystalline TiO_2_ was present between the nanoporous TiO_2_ layer and the ITO surface. Upon illumination electron transfer occurs between Q_A_ and the mesoporous TiO_2_ (1). The function of the blocking layer is to block the interaction of the mobile redox mediator DCBQ (2) with the ITO surface (3). (B) Bar chart showing the mediated and non-mediated photocurrent from the immobilized PSII in the absence and presence of a blocking TiO_2_ layer on the ITO electrode. Photocurrent density recorded from (from left to right) in the absence of the blocking layer, without and with the mediator 100 μM DCBQ and in the presence of the blocking layer, without and with 100 μM DCBQ. Error bars are indicated in gray.

The results indicate that the mediator-induced enhancement of the photocurrent is due to electrons carried from the surface of the nanostructured TiO_2_ to the exposed ITO by the mobile electron carrier. To confirm this model, we studied photocurrents using an electrode in which a layer of crystalline TiO_2_, which is impermeable to any mobile electron carrier in solution (see scheme in Fig. 5A), was used to separate the nanostructured TiO_2_ from the ITO conducting layer. Such layers have been shown to reduce the recombination of the injected electrons and block the interaction between the ITO surface and freely soluble redox active molecules [52]. This electrode structure is expected to give unaltered, or even enhanced, non-mediated photocurrents and to suppress mediated photocurrents. Fig. 5B shows that the separating layer eliminates any enhancement of the photocurrent by DCBQ. This is in good support for the mechanism in which the mediator-induced enhancement (which is only present without the blocking layer) is due to DCBQ acting as an electron carrier shuttling electrons from the TiO_2_ to the ITO.

**Fig. 6:**
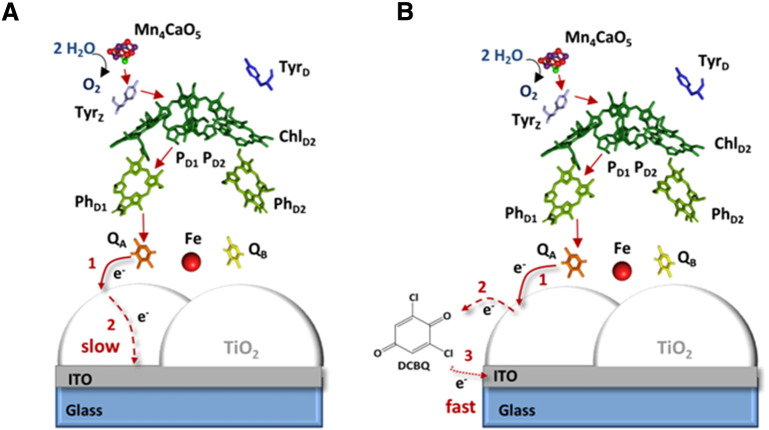
Model describing the electron transfer from PSII to ITO in the absence (A) and presence (B) of the electron mediator DCBQ. (A) Upon illumination electron transfer occurs from Q_A_ to the TiO_2_ (1) and slow electron transfer occurs on the TiO_2_ surface (2) due to the poor conducting properties of the material at an applied bias of + 644 mV *vs* SHE. (B) The redox mediator DCBQ provides an alternative pathway for the electrons by picking up electrons from the TiO_2_ surface (2) and transferring them directly to ITO (3).

All results can therefore be explained by a model describing two situations. In the first case, when no mediator (DCBQ) is present, electron transfer through the metal oxide is slow and the magnitude of the photocurrent may also depend on losses of electrons (for example to oxygen) on the metal oxide surface (Fig. 6A). In the second case, in the presence of the mediator, the electrons that are transferred from the Q_A_ site to the TiO_2_ surface react rapidly with the freely diffusing DCBQ which then delivers them to the ITO. By by-passing the slow electron mobility through the nanoporous metal oxide, DCBQ provides an alternative and more rapid route for the electrons to reach the electrode (Fig. 6B).

The position of the conduction band of TiO_2_ at pH 6.5 is reported to be approximately − 450 mV *vs* SHE [53]. We confirmed this value in our experimental conditions using electrochemical impedance spectroscopy (data not shown). The reduction potential of the Q_A_/Q_A_^−^• couple has been measured to be either − 80 mV [54], [55] or − 140 mV [56], [57] *vs* SHE (but see Ido et al. [58]). The photocurrents were recorded by applying a bias potential of + 644 mV *vs* SHE. Given these values and even when band bending [59] is taken into account, it seems likely that the concentration of trapping states would be very low and the electron mobility on the surface of the metal oxide would be slow. Experimental evidence for poor electron mobility in similar conditions has been reported in studies of interfacial electron transfer between proteins and TiO_2_, where horse-heart cytochrome c with a reduction potential of + 250 mV *vs* SHE was immobilized onto mesoporous TiO_2_ films [60]. The electron density of the TiO_2_ electrode was measured as a function of an applied bias and poor conducting behavior was reported above an applied potential of − 300 mV *vs* SHE. This was attributed to the position of the Fermi level, which lies deep within the band gap of the semiconductor at positive applied potentials. This caused a shift in the measured redox potential of the immobilized cytochrome c, indicating that a larger over-potential was needed to transfer electrons from the conduction band of the semiconductor to the electrode.

Measurements of the steady-state photocurrent as a function of applied bias in artificial water-splitting devices, where photocatalysts are adsorbed onto TiO_2_/FTO electrodes (*e.g.*, Zhao et al. [61]), are usually carried out with lower over-potentials due to the high driving force for electron injection from the photocatalyst into the TiO_2_ conduction band. In these conditions the electron mobility in TiO_2_ is not the limiting step. Furthermore, the possibility that the rate limiting step is between TiO_2_ and ITO is highly unlikely since high current densities are routinely achieved using TiO_2_/ITO electrodes (*e.g.* ref. [61] for a recent example).

## 4 Conclusions

By controlling the formation and the thickness of PSII layer immobilized onto TiO_2_/ITO electrodes and by studying the behavior of the photocurrent in the presence and absence of external mediators and an Q_B_-site inhibitor, we have shown that electrons are transferred to the TiO_2_ directly from Q_A_^−^•. This is not unexpected since it is a relatively low potential electron carrier that is close to an exposed surface of the protein and electron transfer by this route has been suggested, though not demonstrated, earlier [30]. Unexpectedly, the rate-limiting step for photocurrent formation is electron transfer through the TiO_2_. Mobile electron carriers (DCBQ, P*p*BQ and quercetin) are able to take electrons from the TiO_2_ to the ITO thereby enhancing the photocurrent.

**Fig. 7:**
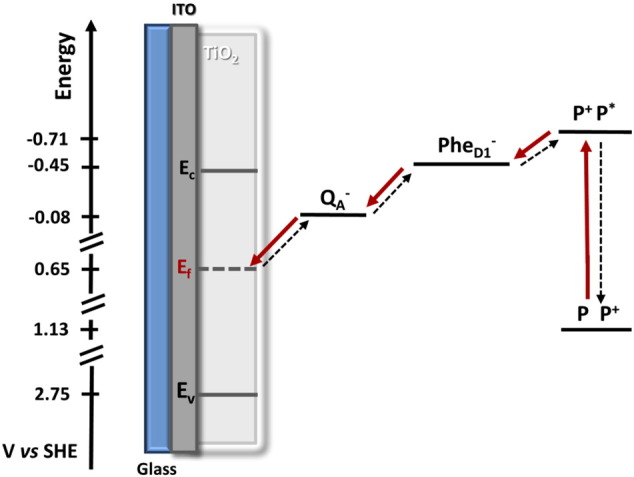
Schematic representation of the electron transfer steps from PSII to the TiO_2_/ITO electrode following the charge separation event and upon the application of a bias potential of + 0.644 V *vs* SHE. The energetic levels of the PSII [62], [58], [63] cofactors involved in the electron transfer process are shown with respect to the position of the conduction band (E_c_), the Fermi level (E_f_) imposed by the bias and the valence band (E_v_) of TiO_2_[64].

The slow rate of electron transfer through the nanostructured TiO_2_ is due to its conduction band (E_c_) being far above both the reduction potential of Q_A_ in PSII (Fig. 7) and also Fermi level (E_f_) which is imposed by the applied bias, resulting in very low electron mobility in the nanoporous TiO_2_. In these circumstance electrons arrive at the semiconductor (in the Fermi level) at an energy level well below the conduction band edge they are thus slow to enter the conduction band if at all. Instead they may remain close to the surface of the material in lower energy states, available for interactions with mediators and slow to migrate to the conducting electrode.

It has been suggested that a driving force of at least ΔG_inj_ = − 0.2 eV is required in order to obtain efficient electron injection from an excited dye-molecule into the conduction band of a semiconductor [65]. It seems likely that a similar requirement will apply to electrons injected from biological systems. It can be seen that even the short-lived Pheophytin anion radical (Phe/Phe^-•^ Em ~ − 500 mV) would be a poor electron donor to TiO_2_.

A more appropriate material for work with PSII should have a conduction band edge at a significantly more positive value. Tin dioxide would appear to be a better candidate given i) its conduction band edge at ~− 200 mV, which is ~ 250 mV more positive than TiO_2_ (Ec ~ − 450 mV) and ii) its better electron mobility. Even so, with the Q_A_ reduction potential being either − 80 mV [54], [55] or − 140 mV [56], [57] *vs* SHE, the driving force is far away from what is required for efficient injection.

Tungsten trioxide appears to be a better candidate, given its conduction band edge at ~+ 50 mV, approximately 200 mV more positive than the reduction potential of Q_A_ and thus nominally conforming to the driving force requirements [65]. The drawback with WO_3_ however is that it has been shown to have poor electron mobility compared to other metal oxides [66].

An alternative that has already been used is the meso-structured ITO, which is a degenerate semiconductor with metal-like conductivity and similar biological compatibility to other metal oxides. Even with this material, however, there are signs of anomalous photocurrent behavior [30], [31], which are likely a reflection of lower than expected conductivity in mesoporous ITO electrodes due to dopant migration in the meso-structured material [67], [68]. It is possible then that some of the limitations seen here for semiconducting TiO_2_ may also apply to meso-structured ITO. We suggest future work with PSII on metal oxides should include better characterization of the electrode material.

The advantage of using transparent mesoporous electrode materials is that they afford the possibility of combining electrochemical and spectroscopic techniques. This broadens the scope for electrochemical studies of the immobilized enzyme thermodynamically and kinetically. The direct electron transfer from Q_A_ to the metal oxide electrode provides a possibility of overcoming turnover rate limitations of the water oxidation due to the slow electron transfer at the PSII acceptor side [69]. For this reason and for any potential applications of these kinds of biohybrid systems, some of the materials mentioned above are worth investigating for use with immobilized PSII.

## Author contributions

The manuscript was written through contributions of all authors. All authors have given approval to the final version of the manuscript.

## Notes

The authors declare no competing financial interest.

## Funding sources

This work was supported by a Biotechnology and Biological Sciences Research Council (BBSRC) grant (BB/K002627/1) and the Royal Society Wolfson Research Merit Award to A. William Rutherford. Florian LeFormal and Li Xiaoe would like to thank the European Science Foundation (project Intersolar 291482) for funding. KB was funded in part by the Heinrich-Böll-Stiftung.

## Transparency document

Transparency document. Image 2

## Appendix A Supplementary data

Supplementary figures. Image 3
