# Supplementary material for: Photocurrents from photosystem II in a metal oxide hybrid system: Electron transfer pathways
Source: Biochim Biophys Acta. 2016 Sep;1857(9):1497–505. doi: 10.1016/j.bbabio.2016.03.004 (PMC4990130; doi:10.1016/j.bbabio.2016.03.004)
Supplement: Supplementary file 2 — Supplementary figures. [file mmc2.docx]

**Supplementary Material**

**Photocurrents from Photosystem II in a Metal Oxide Hybrid System:**

**Electron Transfer Pathways**

*Katharina Brinkert§, Florian Le Formal‡, Li Xiaoe‡, James Durrant‡,*

*A. William Rutherford§* and Andrea Fantuzzi§**

§Department of Life Sciences, Imperial College London, London SW7 2AZ, U.K.

‡Department of Chemistry, Imperial College London, London SW7 2AZ, UK.

Corresponding authors: [a.rutherford@imperial.ac.uk](mailto:a.rutherford@imperial.ac.uk), [a.fantuzzi@imperial.ac.uk](mailto:a.fantuzzi@imperial.ac.uk)

|  |  |
| --- | --- |
|  |  |
|  |  |
|  |  |

**Additional figures**

**
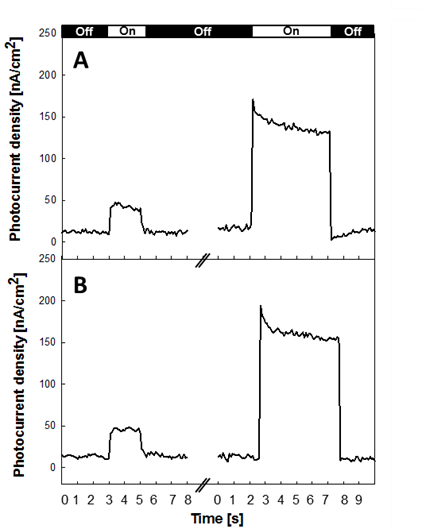
**

**Figure S1**. Photocurrent response from PSII immobilized onto TiO_2_ as a monolayer. (A) 10 μM DCMU was added to PSII prior to its immobilization and photocurrent was measured in the absence (first trace) and presence of 100 μM DCBQ (second trace). (B) 10 μM DCMU was added after the immobilisation of PSII in the absence (first trace) and presence of 100 μM DCBQ (second trace). Note: The bar at the top shows the length of the illumination periods.

**
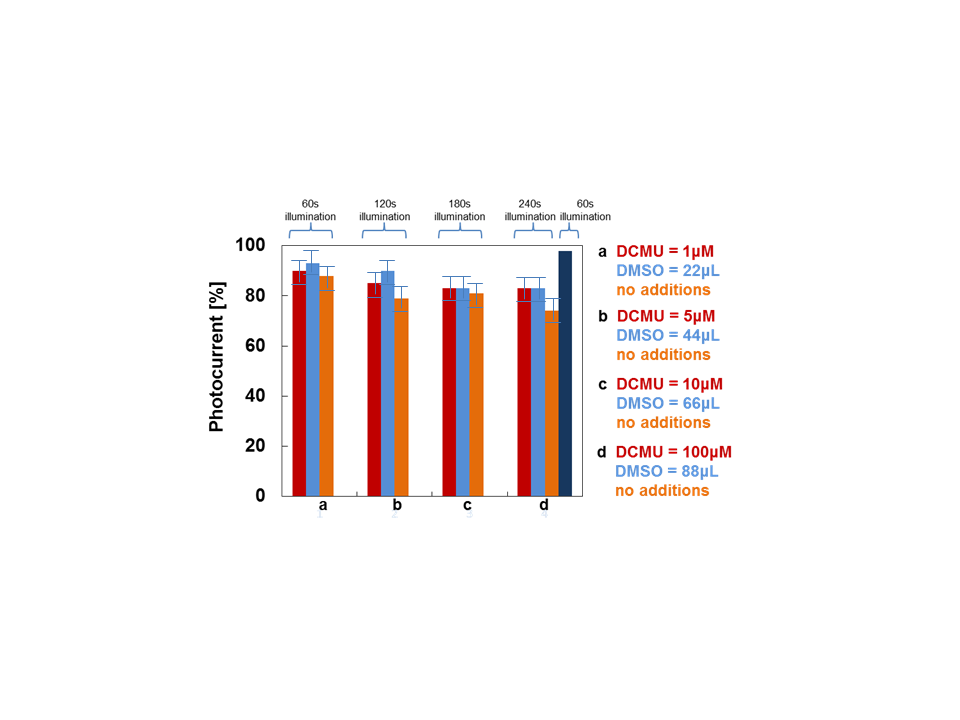
**

**Figure S2.** Influence of increasing concentrations of DCMU and sequential illuminations on the photocurrent. PSII was immobilized onto TiO_2_ as a monolayer and photocurrent measurements were recorded in the presence of 100μM DCBQ. The photocurrent obtained on the first illumination in the presence of DCBQ was taken as 100%. Control experiments in the absence of DCMU are also shown (with and without DMSO, the solvent for DCMU). The results indicate that the PSII monolayer is unaffected by the DCMU treatment and that the insensitivity of the photocurrent is not due to the PSII immobilization since it is unlikely that this affects the binding constant by several orders of magnitude.

The experiments were performed sequentially with increasing concentration of DCMU with the consequence that the PSII was exposed to the light for increasing periods of time as indicated above the bar chart. When compared with a control sample (dark blue bar), which was kept in the dark for 240 seconds and then illuminated for 60 seconds, a small decrease in photocurrent was measured. This small decrease, which was not significantly affected by the presence of DCMU, was attributed PSII photo-degradation.

**
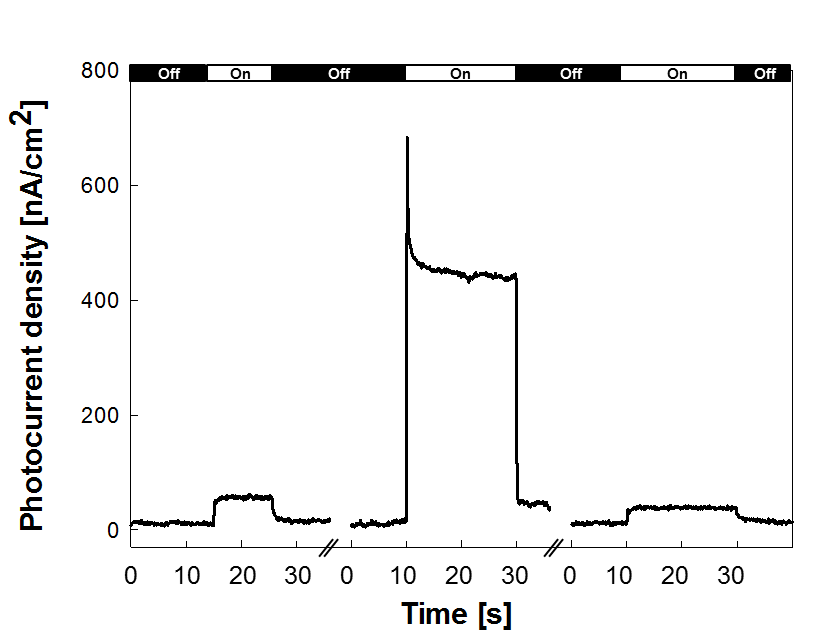
**

**Figure S3.** Photocurrent density from PSII immobilized as a monolayer onto TiO_2_ without mediator (first trace), in the presence of 100 μM DCBQ in measuring buffer (second trace) and after the replacement of the measuring buffer with a fresh solution without DCBQ (third trace). The loss of the photocurrent in the third trace indicates that DCBQ is neither bound to PSII nor to TiO_2_. *Note:* The bar at the top shows the length of the illumination periods.

**Figure S4.** Photocurrent density from PSII immobilized as a monolayer onto TiO_2_ as a function of increasing DCBQ concentrations in the measuring buffer. The maximum photocurrent was recorded in the presence of 100 μM DCBQ. Inset shows the photocurrent recorded for DCBQ concentrations up to 1 μM.
